# Variation of PetCO_2_ during incremental exercise and severity of IPAH and CTEPH

**DOI:** 10.1186/s12890-022-02045-4

**Published:** 2022-06-25

**Authors:** Xingxing Sun, Xue Shi, Yuan Cao, Hanqing Zhu, Bigyan Pudasaini, Wenlan Yang, Ping Yuan, Lan Wang, Qinhua Zhao, Sugang Gong, Jinming Liu, Jian Guo

**Affiliations:** 1 Department of Pulmonary Function Test, School of Medicine, Shanghai Pulmonary Hospital, Tongji University, Shanghai, 200433 China; 2 Department of Respiratory and Critical Care Medicine, Liaocheng People’s Hospital, Liaocheng, 252000 China; 3 Department of Internal Medicine, Columbia Bainuo Clinic, Shanghai, 200040 China; 4 Department of Cardio-Pulmonary Circulation, School of Medicine, Shanghai Pulmonary Hospital, Tongji University, Shanghai, 200433 China

**Keywords:** Pulmonary arterial hypertension, End-tidal PCO_2_, Cardiopulmonary exercise testing, Right heart catheterization

## Abstract

**Background and objective:**

End-tidal PCO_2_ (PetCO_2_) patterns during exercise testing as well as ventilatory equivalents for CO_2_ have been reported for different pulmonary vascular diseases but seldomly for the significant differences in exercise response depending on the etiology of pulmonary hypertension. We aimed to compare PetCO_2_ change pattern in IPAH and CTEPH with varying severity during incremental cardiopulmonary exercise testing (CPET).

**Methods:**

164 IPAH patients and 135 CTEPH patients referred to Shanghai Pulmonary Hospital between 2012 and 2019 were retrospectively recruited into the study. All patients performed CPET and also underwent right-heart catheterization (RHC). Forty-four healthy subjects also performed CPET and were included as controls.

**Results:**

PetCO_2_ was significantly lower in IPAH and CTEPH patients as compared to normal subjects. Moreover, the PetCO_2_ did not rise, in fact fell from rest to anaerobic threshold (AT), then further decreased until peak in both IPAH and CTEPH. PetCO_2_ value at rest, unloaded, AT and peak were proportionately reduced as the World Health Organization functional class (WHO-Fc) increased in both IPAH and CTEPH patients. The PETCO_2_ in IPAH patients had significant differences during all phases of exercise between WHO-Fc I-II and III-IV subgroup. CTEPH also demonstrated significant difference except for PetCO_2_ at peak. PetCO_2_ values were significantly higher in IPAH during all phases of exercise as compared to CTEPH patients (all *P* < 0.001).

PeakVO_2_%pred correlated significantly with PetCO_2_ at rest (r = 0.477, *P* < 0.001), AT (r = 0.609, *P* < 0.001) and peak exercise (r = 0.576, *P* < 0.001) in IPAH. N-terminal natriuretic peptide type-B (NT-proBNP) also correlated markedly with PetCO_2_, with a correlation coefficient of − 0.326 to − 0.427 (all *P* < 0.001). Additionally, PetCO_2_ at rest, at AT and at peak correlated positively with peakVO_2_%pred and showed an inverse correlation with NT-proBNP in CTEPH patients (all *P* < 0.05).

**Conclusions:**

PetCO_2_ during exercise in IPAH and CTEPH patients was significantly different from normal subjects. Moreover, PetCO_2_ values were significantly higher in IPAH during all phases of exercise as compared to CTEPH patients (all *P* < 0.001). PetCO_2_ was progressively more abnormal with increasing disease severity according to peakVO_2_%pred and WHO-Fc.

## Introduction

Pulmonary hypertension (PH) is characterized by a progressive obstructive pulmonary vasculopathy (PV) leading eventually to increased pulmonary vascular resistance and pulmonary arterial pressure and eventually to sustained right ventricular failure and even death [1–3]. PH patients are clinically categorized into 5 groups (Dana Point Classification) [3]. The majority suffer from two distinct key subgroups- idiopathic pulmonary arterial hypertension (IPAH) and chronic thromboembolic pulmonary hypertension (CTEPH) [4, 5].

Though the aetiology of IPAH and CTEPH is clearly different, they closely share pulmonary microvascular histopathological characteristics. The microvascular lesions seen in CTEPH- both occlusive and non-occlusive pulmonary vascular bed, comprising of intimal thickening, plexiform lesions and smooth muscle cell hypertrophy of media, are all considered typical for IPAH as well [6]. The similar progressive PV in both IPAH and CTEPH leads to progressive increases in pulmonary vascular resistance (PVR). The resulting low perfusion of the lungs due to inadequate increase of pulmonary blood flow (cardiac output [CO]) relative to O_2_ exercise demand, leads to an increased ventilation/perfusion mismatch (V/Q) and inefficient gas exchange [5, 7, 8].

Cardiopulmonary exercise test (CPET) has proved to be a potential tool to detect abnormal gas exchange in patients with PH [9]. Dumitrescu et al. [10] described that a high minute ventilation/carbon dioxide output (VE/VCO_2_) value, low end-tidal PCO_2_ at anaerobic threshold (PetCO_2_ @ AT) and abnormal response pattern of PetCO_2_ during incremental exercise can be used to detect the presence of PV from SSc (Systemic sclerosis) patients with normal hemodynamics. The gas exchange parameters during CPET, including peak oxygen uptake (peakVO_2_), VE/VCO_2_ value, physiologic dead space fraction (VD/VTphys), and oxygen uptake efficiency plateau (OUEP) have been validated for grading the severity of disease, to detect PV from early PAH and assess prognosis in patients with IPAH and CTEPH [1, 2, 11–13]. However, the characteristics of PetCO_2_ during exercise in IPAH and CTEPH have rarely been reported.

Our focus therefore was to explore the patterns of PetCO_2_ changes during incremental exercise in IPAH and CTEPH patients, and to potentially correlate PetCO_2_ and severity of the disease.

## Methods

### Subjects

This study retrospectively recruited 299 PAH patients (164 patients with IPAH and 135 patients with CTEPH) who were referred to Shanghai Pulmonary Hospital from 2012 to 2019, and 44 healthy volunteers served as controls. Patients with IPAH and CTEPH all underwent right heart catheterization (RHC) and CPET. In addition, patient demographics, World Health Organization functional class (WHO-Fc), and n-terminal natriuretic peptide type-B (NT-proBNP) were also collected. The study was approved by the Shanghai pulmonary hospital’s ethics committee. Informed consent was obtained from all CPET participants.

IPAH was defined as a mean pulmonary arterial pressure (mPAP) ≥ 25 mm Hg and pulmonary arterial wedge pressure (PAWP) ≤ 15 mmHg with a normal/reduced CO, without known triggering cause. CTEPH was defined as a mPAP ≥ 25 mm Hg and PAWP ≤ 15 mmHg with ventilation/perfusion nuclear medicine scan and/or pulmonary angiogram based on thromboembolic disease [5, 11]. Patients with any evidence of the following: right-to-left intra-cardiac shunt, known coexisting lung diseases (identified clinically or on CT scan), forced expiratory volume in 1 s/percent of predicted forced vital capacity (FEV_1_/FVC%) < 65% or have undergone pulmonary endarterectomy were excluded from the study [2, 14].

### Cardiopulmonary exercise test measurements

All patients and controls performed a symptom-limited ramp-incremental CPET (VIASprint 150 P coupled to a Lab manager CPX cart, CareFusion, Jaeger corp, Hoechberg, Germany) using a breath-by-breath system based on the American Thoracic Society/American College of Chest Physician’s Statement [9, 14]. Before each test, the equipment was calibrated in accordance with manufacturer’s specifications using reference and calibration gases. Standard 12 lead electrocardiograms (ECGs) and pulse oximetry were continuously monitored. Arterial blood pressure (BP) was measured every two minutes with an automatic cuff. The exercise protocol consisted of three minutes of rest, three minutes of unloaded pedaling at 55–65 revolutions per minute, followed by a gradually increasing workload of 5 to 25 watts (W)/min for PAH patients and normal subjects until the maximum tolerance, and five minutes of recovery [15]. Subjects were encouraged to exercise to the limits of their functional capacities or until the physician stopped the test because of some subjective criteria (dyspnea, chest pain, light-headedness and leg effort scores) and objective criteria (peak HR, RER at peak exercise, VE in percent of MVV, and whether or not a plateau in VO_2_). Most CPET values were reported in absolute terms and normalized to percentage of predicted (%pred). Predicted values were calculated using accepted equations [16]. Direct measurements included load, minute ventilation (VE), oxygen uptake (VO_2_), carbon dioxide output (VCO_2_), anaerobic threshold (AT), PetCO_2_, end-tidal PO_2_ (PetO_2_) and several other parameters such as the heart rate (HR), oxygen pulse (VO_2_/HR), respiratory exchange ratio (RER) and VE/VCO_2_ were obtained. Data were recorded every 10-s. Peak VO_2_ was defined as the highest 30-s average of VO_2_ in the last minute of exercise, and other peak parameters were calculated at the same time. AT was determined by the V-slope method [17]. Lowest VE/VCO_2_ was determined by averaging the lowest consecutive 90 s data points [18].

### Grading disease severity

We adopted the classification Sun and Yasunobu have previously reported [7, 19]. They classified PH patients according to disease severity with respect to reductions in percentage of predicted peakVO_2_ (peakVO_2_%pred): mild (65–79% predicted), moderate (50–64% predicted), severe (35–49% predicted), and very severe (< 35% predicted).

### Statistical analysis

Statistical analysis was performed using SPSS (version 20.0, SPSS, Chicago). Continuous variables are presented as mean ± SD or median and interquartile, and categorical variables were presented as numbers. For continuous variables, the unpaired Student t-test and Kruskal–Wallis test were used to identify difference between IPAH and CTEPH patients. The two-way analysis of variance (ANOVA) was used along with post hoc analysis for comparison between subgroups at each time period. Chi-square test was used to identify differences between IPAH and CTEPH group in categorical variables. Correlations between variables were evaluated by partial correlation analysis. *P* < 0.05 was considered significant.

## Results

The characteristics of IPAH, CTEPH and control groups are shown in Table 1. IPAH patients were younger and overwhelmingly female than CTEPH patients (*P* < 0.05). BMI, WHO-Fc and NT-proBNP were comparable between IPAH and CTEPH patients. The mPAP mean right atrium pressure (mRAP) and PVR in IPAH were significantly higher than those in CTEPH (all *P* < 0.05), while CO and CI was the opposite. The PetCO_2_ at rest, PetCO_2_ at unloaded, PetCO_2_ at AT, peak PetCO_2_ were significantly higher than those in CTEPH (all *P* < 0.05). No differences in PAWP, peak VO_2_ and VO_2_/kg at peak were noted between the two groups.

**Table1:** Demographics, WHO-Fc, NT-proBNP hemodynamics and cardiopulmonary exercise testing in IPAH, CTEPH and Control subjects

	Control	IPAH	CTEPH
Age, years	34.75 ± 13.81	40.01 ± 15.20	59.33 ± 12.64#
Gender, male/female	26/18	50/114	60/75#
BMI, kg/m^2^	23.10 ± 2.67	22.69 ± 3.50	23.35 ± 3.13
WHO-Fc, I-II/III-IV	N	66/98	41/94
NT-proBNP, pg/ml	N	994.89 ± 1094.07	1261.95 ± 1353.18
Hemodynamics			
mRAP, mmHg	N	5.89 ± 4.34	4.31 ± 4.11#
mPAP, mmHg	N	56.02 ± 13.35	45.93 ± 13.33#
PAWP, mmHg	N	7.21 ± 3.69	7.24 ± 3.91
CO, L/min	N	4.46 ± 1.57	4.67 ± 1.40#
CI, L/min/m^2^	N	2.63 ± 0.83	2.86 ± 0.69#
PVR, IU	N	12.96 ± 5.64	8.79 ± 4.17#
*Cardiopulmonary exercise testing*			
PetCO_2_ at rest, mmHg	33.85 ± 4.07	26.78 ± 4.27	24.70 ± 3.97#
PetCO_2_ at unloaded, mmHg	37.33 ± 3.93	27.01 ± 5.36	24.42 ± 5.30#
PetCO_2_ at AT, mmHg	42.77 ± 5.06	26.94 ± 6.11	24.36 ± 5.81#
PetCO_2_ at peak, mmHg	39.58 ± 5.90	24.69 ± 6.49	22.49 ± 6.61#
peak VO_2_, ml/min	2148.17 (1496.67, 2527.58)	714.50 (567.50, 928.08)	727.33 (588.00, 853.00)
VO_2_/kg at peak, mL/min/kg	33.70 (29.35, 36.70)	12.30 (9.81, 15.45)	11.81 (10.32, 13.72)

Data are presented as mean ± SD or median and interquartile #*P* < 0.05 versus IPAH patients using unpaired T test and Kruskal–Wallis test

*BMI* body mass index, *WHO-Fc* World Health Organization functional class, *NT-proBNP* n-terminal natriuretic peptide type-B, *mRAP* mean right atrium pressure, *mPAP* mean pulmonary arterial pressure, *PAWP* pulmonary artery wedge pressure, *CO* Cardiac output, *CI* Cardiac index, *PVR* Pulmonary vascular resistance, *Peak VO*_2_ peak exercise oxygen uptake, *AT* anaerobic threshold, *PetCO*_2_ partial pressure of end-tidal carbon dioxide

CPET parameters, NT-proBNP, and hemodynamics of the different groups were summarized in Tables 2 and 3. IPAH and CTEPH patients were divided according to the severity of reduction in peakVO_2_%pred. We found the PetO_2_ at unloaded, PetCO_2_ at unloaded, PetO_2_ at AT, PetCO_2_ at AT, peak load, peak load%pred, peakVO_2,_ peakVO_2_%pred, peak PetO_2,_ peak PetCO_2_ and NT-proBNP, were progressively more abnormal as the physiologic severity increased not only in IPAH (Table 2) but also in CTEPH patients (Table 3). The peak PetCO_2_ decreased in IPAH and CTEPH as peakVO_2_%pred worsened, except in mild and moderate IPAH patients. mPAP and PVR increased whereas CO and CI decreased with increasing physiologic disease severity only in IPAH patients (Table 2). CTEPH parameters were the same as IPAH, except for CO. However, CO also tended to decrease as peakVO_2_%pred worsened (Table 3).

**Table 2:** CPET parameters, NT-pro BNP and Hemodynamics in normal subjects and IPAH patients classified according to peakVO_2_%pred

IPAH	Control (n = 44)	Mild (n = 9)	Moderate (n = 36)	Severe (n = 68)	Very severe (n = 51)
PetO_2_ at Unloaded, mmHg	108.82 ± 5.09	115.20 ± 3.53*	115.60 ± 5.24*	122.09 ± 5.64*#ф	125.59 ± 4.88*#ф§
PetCO_2_ at Unloaded, mmHg	37.33 ± 3.93	31.06 ± 4.66*	31.29 ± 4.36*	26.01 ± 5.16*#ф	23.33 ± 4.60*#ф§
PetO_2_ at AT, mmHg	103.08 ± 6.22	114.58 ± 5.32*	114.88 ± 5.90*	121.90 ± 5.88*ф	124.11 ± 17.41*ф
PetCO_2_ at AT, mmHg	42.77 ± 5.06	31.67 ± 5.79*	32.29 ± 4.90*	26.09 ± 5.33*#ф	22.61 ± 4.08*#ф§
Peak load, W/min	178.08 ± 55.78	85.18 ± 20.05*	85.92 ± 25.06*	62.29 ± 24.61*ф	52.08 ± 19.13*ф
Peak load, %pred	105.93 ± 22.61	100.06 ± 10.54	72.69 ± 13.49*#	49.08 ± 13.58*#ф	34.91 ± 12.57*#ф§
Peak VO_2,_ L/min	2066.22 ± 613.89	987.48 ± 165.97*	985.88 ± 193.87*	749.84 ± 203.76*ф	565.85 ± 139.89*#ф§
Peak VO_2_, %pred	92.25 ± 9.75	72.24 ± 5.00*	57.34 ± 4.67*#	41.22 ± 4.59*#ф	27.83 ± 3.78*#ф§
Peak PetO_2_, mmHg	116.33 ± 5.11	121.35 ± 7.23	122.41 ± 4.68*	127.88 ± 4.93*#ф	131.34 ± 5.22*#ф§
Peak PetCO_2_, mmHg	39.58 ± 5.90	29.68 ± 7.51*	30.12 ± 5.44*	23.48 ± 5.43*#ф	20.36 ± 4.90*#ф§
NT-proBNP, pg/ml	–	474.44 ± 627.95	255.02 ± 323.14	1076.07 ± 1159.07	1502.36 ± 1120.00
mPAP, mmHg	–	45.50 ± 16.61	50.35 ± 13.43	57.44 ± 11.05	59.60 ± 11.57
CO, L/min	–	5.84 ± 2.05	5.49 ± 1.47	4.30 ± 1.58	3.77 ± 1.00
CI, L/min/m^2^	–	3.70 ± 1.18	3.19 ± 0.72	2.49 ± 0.77	2.30 ± 0.59
PVR, IU	–	8.48 ± 3.88	8.92 ± 3.94	13.55 ± 4.91	15.37 ± 6.15

Table presented as mean ± SD. Data among the groups were compared using one-way ANOVA and Tukey multiple comparison test. ^*^P < 0.05 compared to control. ^#^*P* < 0.05 compared to mild. ^ф^*P* < 0.05 compared to moderate. ^§^*P* < 0.05compared to severe

*Peak VO*_2_ peak exercise oxygen uptake, %*pred* percentage of predicted value, *AT* anaerobic threshold, *PetO*_2_ partial pressure of end-tidal oxygen, *PetCO*_2_ partial pressure of end-tidal carbon dioxide, *NT-proBNP* n-terminal natriuretic peptide type-B, *mPAP* mean pulmonary arterial pressure, *CO* Cardiac output, *CI* Cardiac index, *PVR* Pulmonary vascular resistance

**Table 3:** CPET parameters, NT-pro BNP and Hemodynamics in normal subjects and CTEPH patients classified according to peakVO_2_%pred

CTEPH	Control (n = 44)	Mild (n = 30)	Moderate (n = 47)	Severe (n = 36)	Very severe (n = 22)
PetO_2_ at Unloaded, mmHg	108.82 ± 5.09	119.39 ± 5.84*	123.36 ± 5.67*	125.28 ± 5.28*#	128.19 ± 6.28*#ф
PetCO_2_ at Unloaded, mmHg	37.33 ± 3.93	27.04 ± 5.20*	25.17 ± 4.48*	23.04 ± 5.06*#	20.53 ± 5.01*#ф
PetO_2_ at AT, mmHg	103.08 ± 6.22	117.76 ± 6.94*	123.92 ± 6.02*#	126.26 ± 5.22*#	129.26 ± 5.98*#ф
PetCO_2_ at AT, mmHg	42.77 ± 5.06	28.34 ± 6.15*	24.90 ± 4.81*	22.41 ± 5.08*#	20.28 ± 5.29*#ф
Peak load,W/min	178.08 ± 55.78	69.70 ± 30.41*	66.16 ± 22.85*	59.19 ± 22.05*	45.56 ± 26.88*
Peak load, %pred	105.93 ± 22.61	81.33 ± 25.22*	72.81 ± 17.50*	52.25 ± 14.25*#ф	30.36 ± 15.48*#ф§
Peak VO_2_, L/min	2066.22 ± 613.89	921.24 ± 236.47*	786.22 ± 182.26*	681.24 ± 165.14*#	549.26 ± 161.66*#
Peak VO_2_, %pred	92.25 ± 9.75	73.03 ± 9.03*	56.43 ± 4.63*#	42.31 ± 4.14*#ф	29.59 ± 3.05*#ф§
Peak PetO_2_, mmHg	116.33 ± 5.11	122.73 ± 7.16*	126.28 ± 14.74*	130.06 ± 6.40*#	132.38 ± 6.87*#
Peak PetCO_2_, mmHg	39.58 ± 5.90	26.99 ± 6.53*	22.84 ± 5.61*#	20.71 ± 5.94*#	18.24 ± 5.53*#ф
NT-proBNP, pg/ml	–	484.25 ± 588.64	1094.14 ± 1383.23	1546.67 ± 2131.68	2113.27 ± 1620.82
mPAP, mmHg	–	40.03 ± 11.89	45.54 ± 13.15	48.00 ± 14.44	51.14 ± 11.21
CO, L/min	–	4.88 ± 1.92	4.91 ± 1.14	4.52 ± 1.13	4.11 ± 1.40
CI, L/min/m^2^	–	3.23 ± 0.79	2.95 ± 0.67	2.68 ± 0.56	2.55 ± 0.64
PVR, IU	–	6.55 ± 3.47	8.19 ± 3.71	10.07 ± 4.44	10.76 ± 4.13

Table presented as mean ± SD. Data among the groups were compared using one-way ANOVA and Tukey multiple comparison test. ^*^P < 0.05 compared to control. ^#^ *P* < 0.05 compared to mild. ^ф^*P* < 0.05 compared to moderate. ^§^*P* < 0.05compared to severe

*Peak VO*_2_ = peak exercise oxygen uptake, %*pred* percentage of predicted value, *AT* anaerobic threshold, *PetO*_2_ partial pressure of end-tidal oxygen, *PetCO*_2_ partial pressure of end-tidal carbon dioxide, *NT-proBNP* n-terminal natriuretic peptide type-B, *mPAP* mean pulmonary arterial pressure, *CO* Cardiac output, *CI* Cardiac index, *PVR* Pulmonary vascular resistance

### Change in PetCO_2_ during exercise testing

**Fig. 1:**
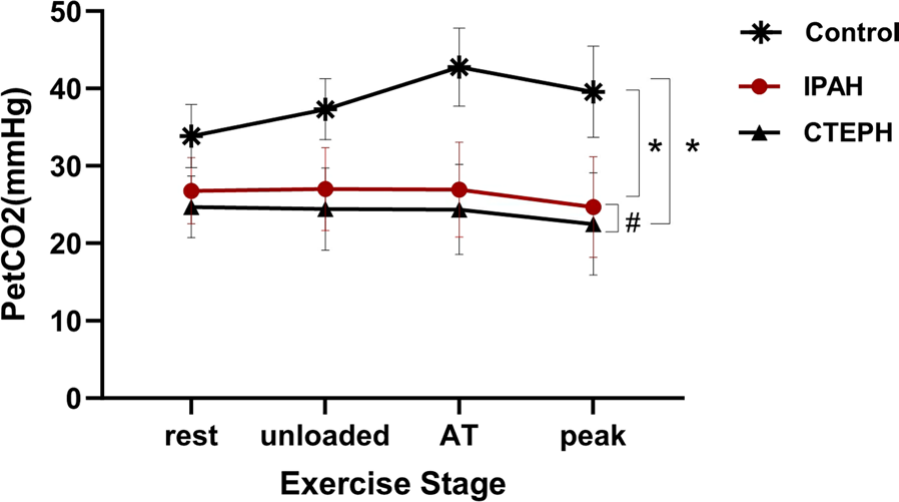
PetCO_2_ differences among the three groups during incremental exercise test. The kinetics of changes in PetCO_2_ among three groups, the mean ± SD of PetCO_2_ in IPAH, CTEPH and control group at different stages of exercise: rest, unloaded, AT, peak are shown in Fig. 1. *P < 0.001, control group versus IPAH group and control group versus CTEPH; # *P* < 0.001, IPAH group versus. CTEPH group

PetCO_2_ patterns during exercise for IPAH, CTEPH and control subjects are illustrated in Fig. 1. In normal subjects, PetCO_2_ distinctively increased during incremental exercise from rest to AT, thereafter decreased only slightly from AT to peak. The pattern of changes in PetCO_2_ was clearly different in IPAH and CTEPH patients, PetCO_2_ did not increase, rather decreased from rest to AT and then slightly decreased until peak, unlike that seen in normal subjects. As expected, IPAH and CTEPH patients had markedly lower PetCO_2_ at four time periods compared with normal subjects (all *P* < 0.001). Although, pattern of changes in PetCO_2_ during exercise was similar in IPAH and CTEPH, PetCO_2_ values were significantly higher in IPAH during all phases of exercise as compared to CTEPH patients (all *P* < 0.001).

**Fig. 2:**
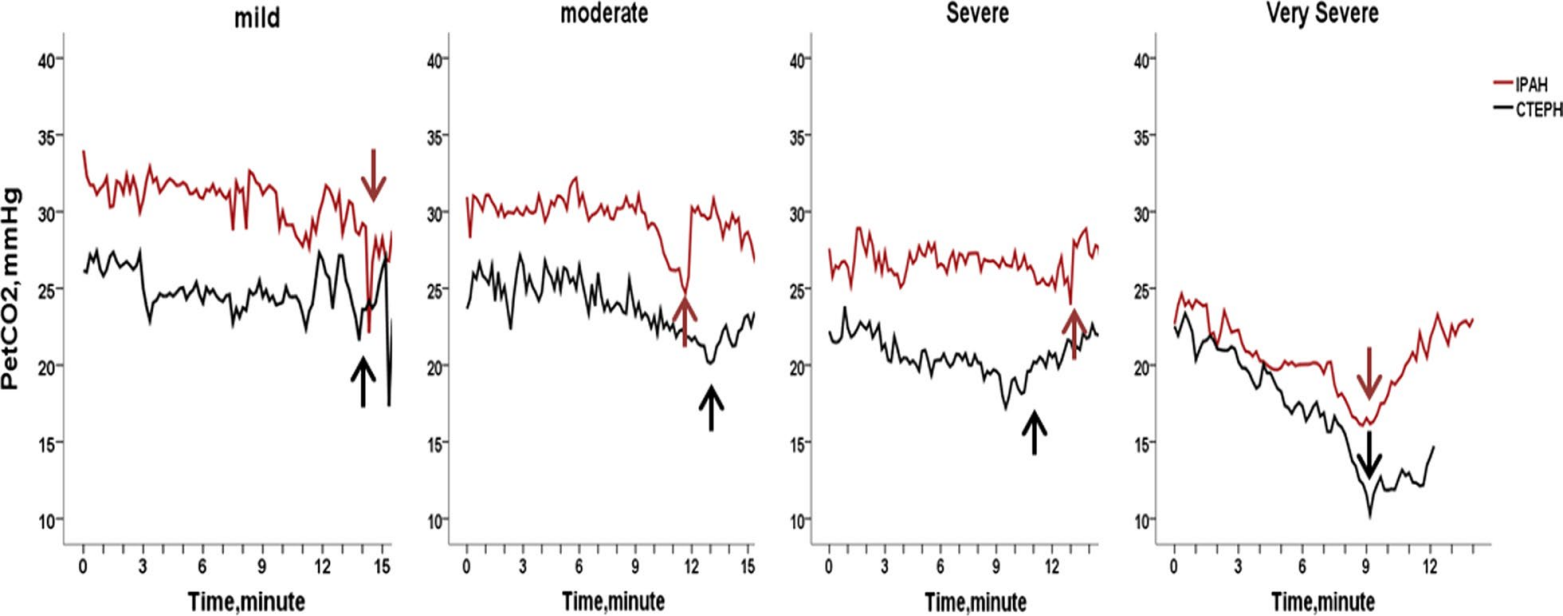
PetCO_2_ patterns during exercise in eight representative subjects with IPAH and CTEPH with relation to severity of exercise limitation. Data were recorded every 10-s. The course of incremental CPET included 3 min of rest, 3 min of unloaded pedaling, followed by a gradually increasing workload exercise until peak, and eventually 3 min of recovery. The red arrows denote peak point in each IPAH subgroup and black arrows is the time of peak in each CTEPH subgroup

**Fig. 3:**
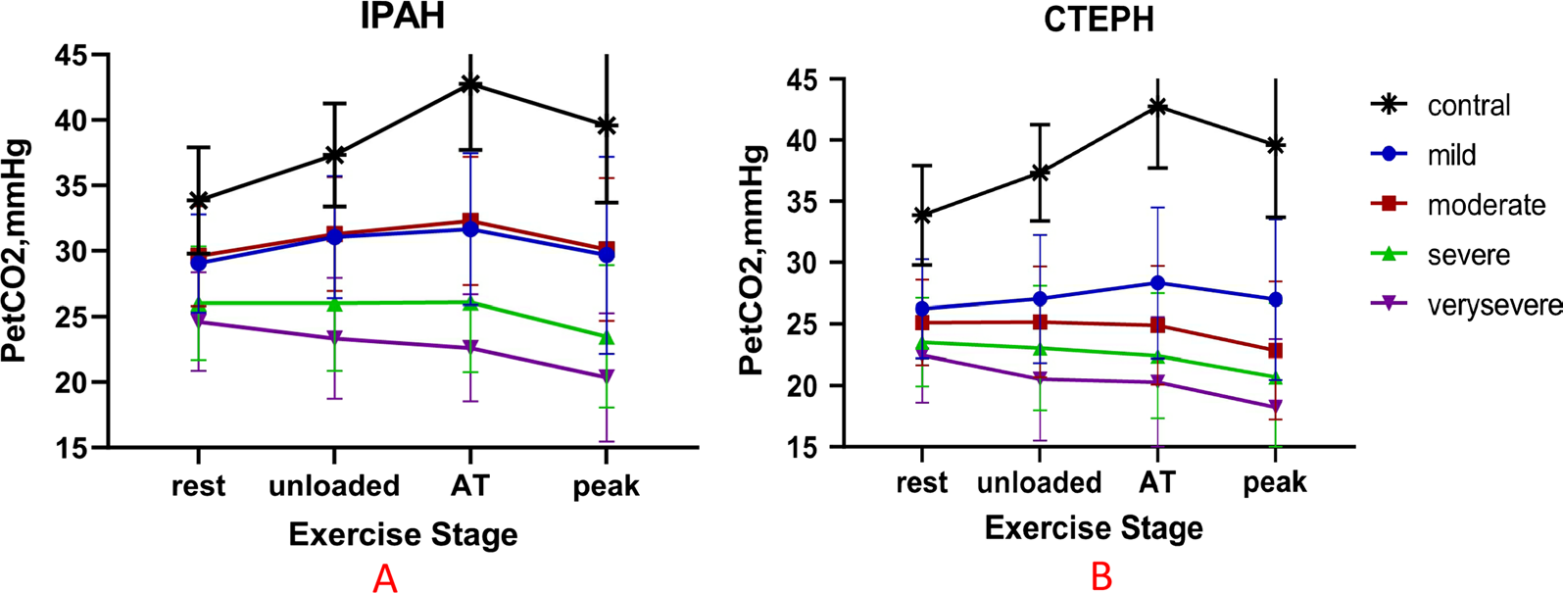
The kinetics of changes in PetCO_2_ during incremental CPET in control, IPAH and CTEPH patients according to disease severity. PetCO_2_ at rest, unloaded, AT, peak were significantly different among each subgroup of IPAH patients. In CTEPH patients, PetCO_2_ were also markedly different among subgroups from unloaded to peak, but no obvious difference was found during rest. Each value is presented as mean ± SD. Statistical analysis was done by two-way ANOVA analysis. **A**: Contral versus mild, moderate, severe, very severe, respectively *P* < 0.05; Mild versus severe, very severe, respectively *P* < 0.05; Moderate versus severe, very severe, respectively *P* < 0.05; Severe versus very severe, *P* < 0.05. **B**: Contral versus mild, moderate, severe, very severe, respectively *P* < 0.05; Mild versus moderate, severe, very severe, respectively *P* < 0.05; Moderate versus severe, very severe, respectively *P* < 0.05

**Fig. 4:**
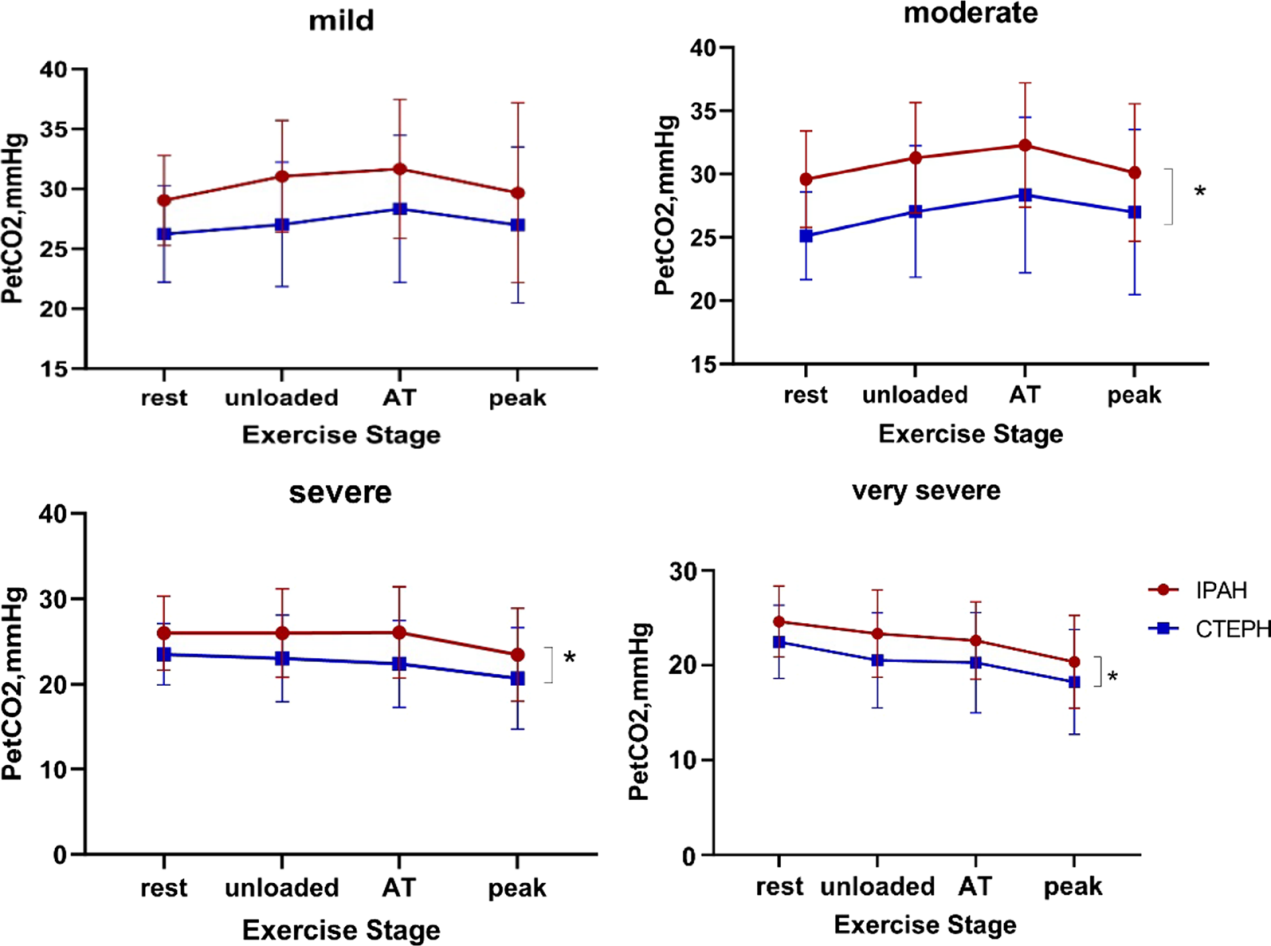
PetCO_2_ differences among IPAH and CTEPH in different disease severity during incremental exercise test. *P < 0.05

PetCO_2_ changes during incremental CPET according to disease severity in IPAH and CTEPH patients are illustrated in Figs. 2, 3 and 4. PetCO_2_ was significantly reduced during at all phases with increased disease severity (all *P* < 0.05) in IPAH and CTEPH, except for mild versus moderate IPAH patients and severe versus very severe CTEPH, in whom the PetCO_2_ values were not clearly different between the two subgroups (Fig. 3). This could be attributed to the smaller sample size of mildly impaired IPAH subgroup. Mild and moderate IPAH patients had only slight increase in PetCO_2_ by approximately 1 mmHg from rest to AT. No noticeable change is seen in severe IPAH patients, in whom PetCO_2_ progressively declined. PetCO_2_ gradually decreased from rest to AT in CTEPH patients. It remained unchanged in mildly impaired CTEPH subgroup (Fig. 3). PetCO_2_ was lower in CTEPH than IPAH at different disease severity during exercise. There was a significant difference in the change of PetCO_2_ between the moderate, severe and very severe groups in IPAH and CTEPH (all *P* < 0.05, Fig. 4). There was no significant difference in PetCO_2_ between the mild groups (*P* > 0.05, Fig. 4).

**Fig. 5:**
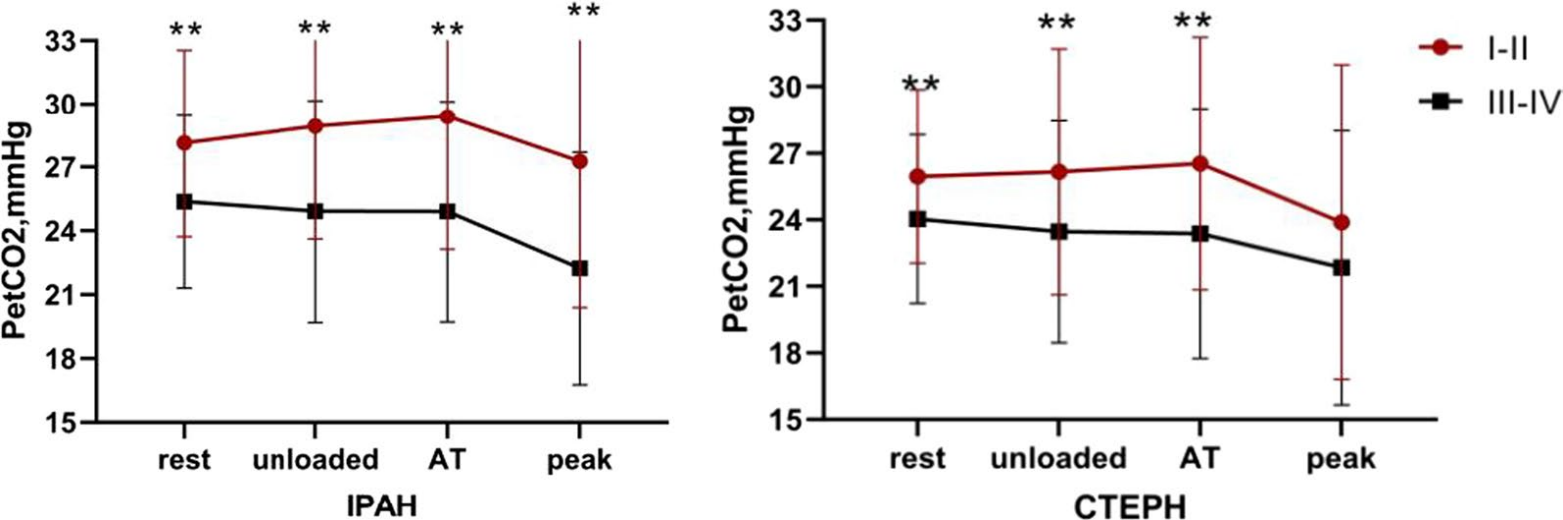
PetCO_2_ at rest, unloaded, AT, peak according to WHO-Fc(I-II and III-IV)in PAH patients. The PetCO_2_ at four time periods were proportionately reduced with increase of WHO-Fc in both IPAH and CTEPH patients. There was a significant difference in PetCO_2_ between WHO-Fc I-II and III-IV group in PAH patients, except for IPAH patients at rest and CTEPH patients at peak. Data are presented as mean ± SD. Statistical analysis was done by unpaired T test, and significant differences between two subgroups at the same time point are shown ** for *P* < 0.01, above value symbol

PetCO_2_ at rest, unloaded cycling, at AT and peak in relation to WHO-Fc (I-II and III-IV) in IPAH and CTEPH patients are illustrated in Fig. 5. IPAH and CTEPH patients were classified into different subgroups based on their WHO-Fc. PetCO_2_ gradually decreased during exercise in both IPAH and CTEPH subgroups, except in WHO-Fc I-II IPAH and CTEPH subgroups, in whom the PetCO_2_ slightly increased from rest to AT (IPAH 28.13 ± 4.41, 28.93 ± 5.31, 29.40 ± 6.26; CTEPH 25.95 ± 3.91, 26.16 ± 5.54, 26.54 ± 5.69) and then decreased until peak (IPAH 27.26 ± 6.87, CTEPH 23.89 ± 7.09). PetCO_2_ values at rest, unloaded pedaling, at AT and peak were proportionately reduced with increase in WHO-Fc in IPAH and CTEPH patients. Significant difference in PetCO_2_ in WHO-Fc I-II and III-IV groups IPAH and CTEPH patients were noted during the four phases of exercise (*P* < 0.01), except at peak in CTEPH.

### Correlations between PetCO_2_ at rest, at AT, at peak, peakVO2%pred and NT-pro BNP

In IPAH patients, PeakVO_2_%pred correlated significantly with PetCO_2_ at rest (r = 0.477, *P* < 0.001), AT (r = 0.609, *P* < 0.001), peak (r = 0.576, *P* < 0.001) and NT-proBNP also correlated markedly with above parameters, with a correlation coefficient of − 0.326 to − 0.427 (*P* < 0.001) (Table 4). In addition, the PetCO_2_ at rest, at AT and at peak correlated positively with peakVO_2_%pred and displayed an inverse correlation with NT-proBNP in CTEPH patients (all *P* < 0.05).

**Table 4:** The PetCO_2_ at rest, AT, peak were correlated with peakVO_2_%pred and NT-proBNP

	PeakVO_2_, %pred		NT-proBNP	
	IPAH	CTEPH	IPAH	CTEPH
PetCO_2_ at rest, mmHg	0.477**	0.434**	– 0.326**	– 0.196*
PetCO_2_ at AT, mmHg	0.609**	0.484**	– 0.427**	– 0.281*
PetCO_2_ at peak, mmHg	0.576**	0.444**	– 0.397**	– 0.263**

*P < 0.05 **P < 0.01

## Discussion

PetCO_2_ was significantly lower in IPAH and CTEPH patients than in normal subjects during the four phases of exercise. PetCO_2_ was higher in IPAH compared to CTEPH, and the pattern was different. While authors from a previous Harbor-UCLA study have reported PetCO_2_ response to exercise in primary pulmonary hypertension (PPH) in a small number of patients [19], ours is the first study about the measurements correlate with severity in a homogenous Chinese patient cohort. Secondly, even decreased from rest to AT and gradually reduced in proportion to the increase in severity of physiologic impairment. Moreover, the PetCO_2_ at different exercise stages were also reduced with an increase in WHO-Fc. Thirdly, PetCO_2_ at rest, at AT and at peak were correlated with NT-proBNP and peak VO_2_. Statistically significant relation was found not only in IPAH but also in CTEPH patients. All of our findings demonstrate the response pattern of PetCO_2_ to be abnormal during incremental exercise, worse in CTEPH patients compared to IPAH. PetCO_2_ potentially can be used as an index to reflect disease severity and heart failure.

The increased ventilation/perfusion mismatch caused by pulmonary vasculopathy in IPAH and CTEPH [2, 5, 11, 19] leads to increased physiological dead space, a lower PetCO_2_ compared with normal subjects in whom ventilation and perfusion are uniformly distributed. While there were no significant differences in WHO-Fc and NT-proBNP between the two groups in our study, CTEPH patients had significantly decreased mPAP, PVR and higher oxygen pulse %pred. This indicates that CTEPH patients may have better cardiac reserves and milder PV than IPAH. However, we also found CTEPH to have increased ventilatory inefficiency and lower PetCO_2_ compared with IPAH, which may indicate to a more proximal vascular occlusion in CTEPH than in IPAH. This leads to the VD/VT and ventilation drive to be higher in CTEPH than IPAH, which corroborates the finding from an earlier study [2], that reported a higher OUEP and better ventilatory efficiency in IPAH than in CTEPH.

PetCO_2_ values distinctively increased during incremental exercise from rest to AT and respiratory compensation, thereafter, it decreased until peak in normal subjects [20, 21]. The PetCO_2_ in Cardiac Patients was below normal during incremental exercise, and markedly reduced with increase in NYHA functional class [21], yet the response patterns of PetCO_2_ from rest to AT between Cardiac Patients and normal subjects were similar. Tanabe et al. [22] report that in patients with chronic heart failure, the pattern of changes in PetCO_2_ during exercise was normal even in NYHA class III patients. In our study, we found that IPAH and CTEPH patients not only had reduced PetCO_2_ in proportion with the increases in disease severity and WHO-Fc, but also exhibited an abnormal pattern of change in PetCO_2_ i.e., PetCO_2_ remained unchanged, rather decreased from rest to AT and further declined until maximal exercise. This abnormal pattern of PetCO_2_ may be attributed to a higher VD/VT in IPAH and CTEPH than that in chronic heart failure (CHF) patients. In CHF patients, increased ventilatory drive have been explained by increased dead space relative to low CO, early lactic acidosis, increased chemosensitivity due to increased sympathetic tone [21, 23–25]. Additional factors in IPAH and CTEPH could include pulmonary vascular obliteration [2, 5, 11], and hypoxemia due to a right-to-left shunt [14, 19], that leads to a greater increase of VD/VT which further increases with exercise.

VO_2_max is the best index of aerobic capacity and the gold standard for cardiorespiratory fitness. In chronic pulmonary vascular disease, the VO_2_max provides an index of severity and significantly correlates with the amount of functional vascular bed. It is lower in patients with high PVR and low cardiac index (CI) [9]. Peak VO_2_ often serves as an estimate for VO_2_max. Additionally, CPET-derived peakVO_2_ has a prognostic significance that is superior to resting haemodynamic parameters in both IPAH and CTEPH patients [1, 26]. So based on the study by Yasunobu and Sun [7, 19], we divided the patients with IPAH and CTEPH into different groups of disease severity according to reduction in peak VO_2_%pred. PetCO_2_ gradually increased only about 1 mmHg in mild and moderately impaired IPAH group with exercise. It remained unchanged in severely impaired group and progressively decreased from rest to AT in very severely impaired group. In IPAH patients, there was no clear difference of PetCO_2_ at four time period between mild and moderate subgroup, probably due to the small sample size of mildly impaired IPAH group. The value of PetCO_2_ in mild and moderate subgroups in IPAH and CTEPH was markedly lower than that in normal subjects, but the pattern of changes in PetCO_2_ between two subgroups and normal subject were similar, which is inconsistent with Yasunobu’s study, in which they report that PetCO_2_ remained unchanged in mild subgroup and gradually decreased in moderate subgroup from rest to AT. In addition, our study showed that PetCO_2_ remained unchanged from rest to AT in the IPAH severe subgroup, in contrast to their patient subgroup, in whom PetCO_2_ progressively decreased during exercise. This discrepancy may be attribute to: Sun et al. grouped the PPH patients into 4 groups on the basis of reductions in PeakVO_2_%pred. Their study showed that the hemodynamic severity was gradually increased as the physiological severity advanced (mPAP was 48 ± 17 mmHg, CO was 5.1 ± 1.1 L/min, PVR was 8 ± 4 mmHg/L/min in mild subgroup, mPAP was 63 ± 14 mmHg, CO was 4.4 ± 1.4L/min, PVR was 15 ± 8 mmHg/L/min in moderate subgroup, mPAP was 70 ± 18 mmHg, CO was 3.5 ± 1.0 L/min, PVR was 18 ± 5 mmHg/L/min in severe subgroup). Compared with their study, the IPAH patients in our center also had progressively abnormal hemodynamic index with an increase of physiological severity, yet the mild, moderate, severe subgroup in IPAH had lower mPAP, PVR and higher CO. Which indicates that PPH patients from the Harbor-UCLA study may have had worse PV than those from our center even in the same physiological severe class. IPAH patients in our center had almost normal patterns of changes in PetCO_2_ in mild to moderate subgroup and better response patterns of PetCO_2_ to exercise in severe subgroup than their (Harbor-UCLA) patient subgroup. At this point it is speculative to suggests that PV in a predominantly Asian population was milder than those in a predominantly Caucasian population at the same peakVO_2_%pred range. This finding is perhaps owing to the younger age group of our IPAH cohort. Additionally, less than 4% of hospital diagnosed PH cases in China was classified as idiopathic in a large surveillance study [27]. These ideas regarding reduction in PeakVO_2_ need further validation as there is paucity of clear data regarding PH in China. With the lack of large national epidemiological study or a true national registry the extent of PH and its clinical implication in a huge country like China is a drawback. Moreover, like in other developed nations the prevalence of PH from all causes is increasingly being reported, yet what is the extent of PH prevalence in China we simply do not know. PH from all causes may be potentially affecting millions of people in China.

Dumitrescu et al. [10] showed that PV impairs dilatation of affected pulmonary blood vessels, impeding pulmonary blood flow during exercise. This leads to an increased ventilation-perfusion mismatch and abnormal gas exchange, findings that are characteristic of ventilation-perfusion mismatch. Thus, patients with PV would display inefficient gas exchange, especially reduced PetCO_2_ and abnormal patterns of PetCO_2_ changes during exercise. Galiè et al. [6] described that the distal pulmonary vasculopathy in CTEPH, both in occluded and non-occluded pulmonary vascular bed, is characterized by lesions considered typical for idiopathic pulmonary arterial hypertension, including plexiform lesions. Due to similar pulmonary vasculopathy in patients with IPAH and CTEPH, we hypothesized CTEPH patients to have abnormal pattern of changes in PetCO_2_ during exercise. In the present study, we found that the patients with CTEPH also have reduced PetCO_2_ at rest and during exercise, with PetCO_2_ decreasing with increase in physiologic disease severity and WHO-Fc. Additionally, abnormal pattern of changes in PetCO_2_, i.e. PetCO_2_ gradually declined during incremental exercise, except for CTEPH mildly impaired subgroup, whose PetCO_2_ remained unchanged from rest to AT and decreased until peak was noted. The value of PetCO_2_ and the pattern of its changes during exercise appear to reflect physiologic disease severity and degree of heart failure in CTEPH patients.

In chronic pulmonary vascular disease, the VO_2_max provides an index of disease severity [9]. In addition, peakVO_2_ has a prognostic significance superior to most standard resting haemodynamic parameters in IPAH and CTEPH patients. NT-proBNP and WHO-Fc are also commonly used clinical parameters to predict prognostic information for PH patients [1, 19, 28–30]. In the present study, we found that the PetCO_2_ reduction correlated with an increase in WHO-Fc not only in IPAH patients but also in CTEPH patients. We also found that PetCO_2_ at different exercise stages were significantly correlated with peakVO_2_%pred and NT-proBNP. In turn parameters that were correlated with peakVO_2_%pred and NT-proBNP appear more significant with increased exercise intensity in both IPAH and CETPH. This indicates that PetCO_2_ can be a reflection of the prognosis to a certain extent in the two forms of PH.

The drawbacks in our study include, firstly, this was a retrospective analysis with demographic differences with age and gender between the two disease subtypes. Secondly, due to the small sample size and highly selective patient population, the value of PetCO_2_ to reflect disease severity and prognosis remains to be proven by studies which are prospective and have generally more inclusive PH patients. In addition, the arterial blood sample was not measured dynamically, which would have provided better gas exchange data during exercise and better evaluation of chemosensitivity.

## Conclusion

Compared with normal subjects, the PetCO_2_ in both IPAH and CTEPH patients at rest and during exercise were markedly reduced and progressively decreased with increased disease severity. The response pattern of PetCO_2_ during exercise in the two IPAH and CETPH groups was significantly different from that in normal subject, except in mild and moderate IPAH patients. Additionally, PetCO_2_ at different exercise stages in both groups significantly correlated with peakVO_2_%pred and NT-proBNP. These findings are promising and indicate that PetCO_2_ could be an index reflecting disease severity and prognosis.

## Data Availability

All the related data are presented in the manuscript.

## Abbreviations

IPAH: Idiopathic pulmonary arterial hypertension
CTEPH: Chronic thromboembolic pulmonary hypertension;
PH: Pulmonary hypertension
PV: Pulmonary vasculopathy
PetCO_2_: End-tidal partial pressure for carbon dioxide
CPET: Cardiopulmonary exercise testing
VE/VCO_2_: Minute ventilation/carbon dioxide output
BMI: Body mass index
RHC: Right heart catheterization
V/Q: Ventilation/perfusion scan
CI: Confidence interval
WHO-FC: World Health Organization functional class
NT-proBNP: N-terminal natriuretic peptide type-B
mPAP: Mean pulmonary artery pressure
PAWP: Pulmonary arterial wedge pressure
CO: Cardiac output
CI: Cardiac index
PVR: Pulmonary vascular resistance
mRAP: Mean right atrial pressure
VO_2_: Oxygen uptake
VCO_2_: Carbon dioxide output
VD/VT: Dead space ventilation as a fraction of tidal volume
PETO_2_: End-tidal partial pressure for oxygen
VCO_2_: Carbon dioxide output
AT: Anaerobic threshold
OUEP: Oxygen uptake efficiency plateau
SSc: Systemic sclerosis
FEV_1_/FVC%: Forced expiratory volume in 1 s/percent of predicted forced vital capacity
ECGs: Electrocardiograms
BP: Blood pressure
HR: Heart rate
VO2/HR: Oxygen pulse
RER: Respiratory exchange ratio
PPH: Primary pulmonary hypertension
CHF: Chronic heart failure
